# Patient-centeredness—a cultural targeted survey among junior medical managers

**DOI:** 10.1186/s12939-023-01979-3

**Published:** 2023-08-30

**Authors:** Orna Tal, Royi Barnea, Aviad Tur-Sinai

**Affiliations:** 1 Shamir Medical Center, Be’er Yaakov, Israel; 2 Bar-Ilan University, Ramat Gan, Israel; 3 ICET, Israeli Center for Emerging Technologies, Beer Yaakov, Israel; 4 Assuta Health Services Research Institue, Assuta Medical Centers, Tel Aviv, Israel; 5 School of Health Systems Management, Netanya Academic College, Netanya, Israel; 6 Department of Health Systems Management, The Max Stern Yezreel Valley College, Yezreel Valley, Israel; 7 School of Nursing, University of Rochester Medical Center, Rochester, NY USA

**Keywords:** Patient-centeredness, Equity, Junior medical managers, Culture, Standpoints, Values

## Abstract

**Background:**

Patient-centeredness is a core element in healthcare. However, there is a gap between the understanding of this term by healthcare professionals, and patients’ capability, self-efficacy, and willingness to take part in medical decisions. We aim to expose standpoints toward “patient centeredness” among junior medical managers (JMM), as they bridge between policy strategies and patients. We try to reveal cultural differences by comparing the views of the majority and the minority subpopulations of Israel (Arabic and Hebrew speakers).

**Methods:**

A cross-sectional survey among JMM studying for an advanced degree in health-system management at three academic training colleges in Israel was conducted in February–March 2022. The respondents completed a structured questionnaire comprising four sections: a) perceptions of trust, accountability, insurance coverage, and economic status; b) perceptions regarding decision-making mechanisms; c) preferences toward achieving equity, and d) demographic details.

**Results:**

A total of 192 respondents were included in the study—50% Hebrew speakers and 50% Arabic speakers. No differences were found between Arabic and Hebrew speakers regarding perception of trust, accountability, insurance coverage, and economic status. JMM from both subpopulations believed that patients’ gender and age do not influence physicians’ attitudes but Arabic-speaking respondents perceived that healthcare professionals prefer educated patients or those with supportive families. All respondents believed that patients would like to be more involved in medical decisions; yet Arabic-speakers perceived patients as tending to rely on physicians’ recommendations while Hebrew speakers believed that patients wish to lead the medical decision by themselves.

**Conclusions:**

Patient-centeredness strategy needs to be implemented bottom-up as well as top-down, in a transparent nationwide manner. JMM are key actors in carrying out this strategy because they realize policy guidelines in the context of social disparities, enabling them to achieve a friendly personalized dialogue with their patients. We believe that empowering these JMM may create a ripple effect, yielding a bottom-up perception of equity and initiating change.

## Background

Patient-centeredness is a leading core element in healthcare [1]. However, there is a gap in understanding this term between healthcare professionals providers and therapists and patients’ capability, self-efficacy and willingness to take part in medical decisions [2, 3].

Health literacy (HL) is the knowledge and competence to access, understand, appraise, and apply health information to health judgment [4]. HL can help individuals to achieve more control over their health and over the determinants of health [5] and, thus, to make appropriate health-related decisions wisely. Several studies have demonstrated a meaningful relationship between HL and health behaviors [5, 6]. Education level, socioeconomic status (SES), and physical limitations are found to make the strongest contributions to inadequate or problematic HL [4]. It has been shown that in Israel SES and the living environment make a relatively high and substantial contribution to self-rated health, followed by psychosocial factors and cultural health habits. Therefore, ethnic differences between Arabs and Jews yield health inequalities beyond personal characteristics [7].

Patients obtain information about their medical condition from multiple sources including scientific papers, health organizations’ websites, patient associations’ websites, and social media [8, 9]. Although the availability of information intended for a lay audience from reliable sources has been growing, including side effects of treatment [10], risk factors [11], and other health-related parameters [12], there is no evidence on how patients interpret or understand its implications. Moreover, patients’ prioritization of different information aspects has not been investigated; thus, we still lack patients’ perspective on the value of data. (For example, what do patients look for first, safety of treatment or novel approaches to coping with their condition?)

Adherence to treatment is a crucial component in achieving quality of care and successful medical treatment as well as good health outcomes [13–15]. A recent systematic review pointed to the significance of interpersonal communication and counseling- and education-based interventions for patient adherence to treatment [16]. Adherence to treatment also depends on various parameters such as patient’s age, education, ethnicity (especially among minorities), and medical condition [16–18].

Shared Decision Making (SDM) is a process by which a medical choice is made by the patient, his/her significant others, or both, together with one or more healthcare professionals [19]. SDM has been recognized as a useful tool for improving prudency in healthcare and has been linked to self-efficacy and empowerment of service users [20]. A recent study has demonstrated that lack of information and negative experiences reported by friends or family members affect the decision-making of parents who are asked to vaccinate their children against human papilloma virus [21].

Although physicians are expected to treat all patients equally, patients’ personal characteristics and medical professionals’ personal communication skills, culture, values, and preferences may influence the patient–caregiver relationship. Patients’ educational background and language proficiency are key elements in establishing good patient–physician communication. A study conducted in 31 countries showed that patients with lower language proficiency are more likely to experience more negative interactions with their physicians, while highly educated patients are more likely to experience positive patient–physician interaction [22].

Given the paucity of resources in public healthcare systems, physicians must often consider costs when making decisions about appropriate care. Medical managers at all levels constantly face the need to prioritize medical services by allocating resources to selected treatments, emphasizing a major ethical dilemma [23]. Some suggest that stakeholders should be regarded as a “social market” of providers and buyers, existing in constant tension that leads to patient decisions that weigh social forces against economic barriers [24].

Prioritizing medical treatment from the physician’s point of view is relevant to the medical settings, the severity, or the prevalence of a medical condition and the patient’s personal predisposing characteristics (such as age [25], gender, or survival odds). Socio-ethical values, such as allocation of massive resources to only a few [26] or the patient’s former contribution to society, also arise.

Furthermore, economic considerations may play a role in choosing healthcare services, involving both patient spending choices and preferences, and organizational or national priority setting. Although the Israeli healthcare system is publicly funded, the country’s emerging private healthcare market is changing both patients’ and physicians’ awareness of the possibility of purchasing healthcare services, thus also taking patients’ willingness to pay into consideration. Moreover, physicians may consider the patient’s type of insurance, as in Israel more than 80% of the population purchases supplemental health insurance and 40% have private coverage, expanding the possibilities of care beyond the basic publicly funded health insurance [27, 28] and allowing for out-of-pocket expenses and reimbursement [29]. Employment status may also have some effect on perceptions of health equity and gaps [30].

Although many healthcare systems have been recently evoking the need to expand patient-centeredness as a socio-ethical and clinical value, the common trend is to start infiltrating the idea top-down, meaning that this strategy still needs to gain access to caregivers and then to patients and the general public. In this study, we wished to investigate whether this strategic concept has already been implemented and accepted by healthcare professionals who work with patients daily. To answer, we focused on junior medical managers (JMM), who bridge between policymaking strategies and actual issues and patients’ self-reported experience. Moreover, we assumed that the perception of inequity may be driven by JMM themselves because they are influenced by their own access to care, primary physicians, and other facilities on the basis of geographic distance or place of residence (rural vs. urban areas) and their cultural background. Finally, we wanted to know whether the social environment and tradition of JMM themselves play a role in their perception of equity. Thus, we chose to sort them into two subgroups based on their declared spoken language rather than religion or place of residence.

The attitude of healthcare professionals and providers toward including patients as partners in the decision-making process may have a significant influence on patients’ perception as “being in the center of care” [31]. Therefore, it is essential for policymakers to understand the perceptions of healthcare-system workers [32]. Discovering the standpoints of JMM is especially interesting because JMM can point to gaps and barriers in the implementation of health equity and the patient-centeredness approach in the actual patient–physician encounter. Therefore, in this study we aimed to examine the standpoint of JMM towards patient centeredness and to analyze the main leading elements in their professional perception. As the population of Israel comprises two major ethnicities—Arabs and Jews—we also asked whether professional, personal, or cultural attributes underlay differences between their standpoints. This comparison of Jewish and Arab healthcare professionals may shed light on their conceptions of health inequality and its effect on patients. Research on these gaps among healthcare workers and, particularly among future managers of the healthcare system, is, however, deficient.

This point is especially interesting in view of previous reports of perceptions of unfairness among the Arab population in Israel, whereas the Jewish population did not report any discrimination or stigma against Arabs [33]. Cultural differences and similarities between Jews and Arabs with regard to coherence and hope have also been demonstrated [34].

## Method

### Setting and participants

We conducted a cross-sectional survey among JMM (mostly physicians and nurses) studying for an advanced degree in health management at three academic colleges located in the northern, western, and central regions of Israel.

The JMM sample was chosen for two reasons: 1) to target healthcare workers who encounter patients on a daily basis but are also familiar with regulations and policy, and 2) to examine the differences between the minority (Arabic-speaking) and majority (Hebrew-speaking) populations in Israel, which also represent the general population.

As we specifically aimed to examine the standpoints of the Arab population—an ethnic minority constituting 21% of the Israeli population [35]—the target population was a corrected sample that included 50% Hebrew-speaking participants and 50% Arabic-speaking participants. We considered native spoken language rather than religion because we feel that this may be considered as a better marker of culture.

The study was approved by the institutional ethics committee (ASF 008–22). The participants freely volunteered to respond to the questionnaire. All personal details were removed from the dataset. To comply with the anonymity requirement, the participants’ personal details were omitted from the final version.

### Questionnaire and data collection

The questionnaire comprised four sections: a) general perceptions of trust, accountability, insurance coverage, and economic status (six questions), b) participants’ perceptions of the decision-making mechanism (four scenarios, detailed in the appendix), c) scoring the relative importance of elements that affect equity (seven items), and d) demographic details such as profession, experience, place of residence and work, age, gender, and main spoken language. The items were constructed from the viewpoint of a public healthcare provider who administers a wide range of essential health services and considers equity a core value of the Israeli public health system (Table 1).

**Table 1:** Perception of Junior Medical Managers on patient and physician trust, accountability, insurance coverage, economic status

			Population			
Confidence in enabling provision of care	Parameter	Scale	Total (%)	Hebrew-speaking participants (%)	Arabic-speaking participants (%)	P value
Trust	Confidence in receiving best care	1–10	6.65	6.44	6.73	0.198
	Equity in access to care	1–10	5.83	5.89	5.80	0.756
	Confidence in being able to take care of own health*	1–4	2.32	2.34	2.34	1.000
	Physician’s accountability to patient’s adherence to care	1–10	8.84	8.92	8.73	0.432
Economic considerations	Physician’s awareness of patient’s insurance coverage	1–10	4.9	4.53	5.39	0.057
	Physician’s awareness of patient’s economic status	1–10	5.39	5.17	5.97	0.089

The data were collected in February and March 2022. The questionnaire was filled in by using a convenient sample. A link to the electronic form of the questionnaire was sent to the participants. The response rate was approximately 50% of the study sample.

### Statistical analysis

The data were analyzed descriptively. Categorical variables were summarized by number and percentage and compared by chi-squared test. Continuous variables were summarized as mean with standard deviations (SD) and compared by t-test for independent samples. P values smaller than 5% were considered statistically significant. In addition, to explain perceptions of components of trust, an econometric model was used to estimate the difference between Jewish and Arab junior medical managers.

## Results

### Descriptive statistics

A total of 192 JMM (71% of them women) completed the questionnaire: 96 (50%) Hebrew speakers and 96 (50%) Arabic speakers. The respondents’ average age was 37.7 years (SD 8.9). Most respondents (71.4%) were nurses, 6.8% were physicians, and the rest were other healthcare professionals. The respondents’ average seniority in the healthcare system was 11.5 years (SD 8.9).

Women comprised 79.8% of Hebrew-speaking respondents as against 61.5% of Arabic-speaking respondents (p < 0.01). Hebrew speakers were statistically significantly older than Arabic speakers (40.9 [SD 1.0] years vs. 34.4 [SD 0.7] years, p < 0.001) and had longer seniority in the healthcare system (13.7 [SD 1.0] vs. 9.3 [SD 0.7], p < 0.001). The percentage of nurses was higher among Arabic-speaking respondents than among Hebrew-speaking respondents (81.3% vs. 61.5%), while the percentages of healthcare-system workers and medical doctors were lower (13.5% vs. 27.1 and 3.1% vs. 10.4%, respectively).

We present the results in three layers: a) general perception of national ethical values, b) the patient–physician relationship, and c) the relative importance of different elements and their impact on the attainment of equity.

#### General perception of national ethical values

Two major values were compared: trust, reflected in confidence in enabling the provision of care, patient–physician trust, accountability, and insurance coverage; and the ability to meet demand, reflected in economic status. The results are presented in Table 1.

Two items aimed to reveal the perception of JMM on patients’ trust in the healthcare system. The respondents ranked trust at 6.65 (on a 1–10 scale) and equity at 5.83. No statistically significant differences between the subpopulations of respondents were observed.

Analyzing the levels of trust, the respondents perceived that patients trust physicians more (8.84 on a 1–10 scale) than they trust the healthcare system (6.65), while their confidence in being able to take care of their own health is only modest (2.34 on a 1–4 scale). No statistically significant differences between the Hebrew- and Arabic-speaking populations were observed.

Another aspect investigated was the economic perspective because it may also be a barrier to access to care. Two items evaluated the perceived importance of patients’ **economic status** from physicians’ perspective (Table 1). Both were ranked relatively low with a variation in trends between the subpopulations: The importance of having additional (supplemental / private) insurance was ranked at 4.9 (4.53 by Hebrew speakers vs. 5.39 by Arabic speakers, p = 0.0575). The second question, “How important is it for the physician to be aware of the patient’s economic status?” was ranked at 5.39 (5.17 by Hebrew speakers and 5.97 by Arabic speakers, p = 0.0898).

#### Patient–physician relationship and decision-making

JMM’s perceptions of elements of patients’ abilities to deal with medical decisions are presented in Table 2.

**Table 2:** Junior Medical Managers’ perception of the decision-making mechanism by scenario and subpopulation

		Population			
Mechanism	Who is leading the process?	Total (%)	Hebrew-speaking respondents (%)	Arabic-speaking respondents (%)	P value
Decision-making process	**Physician** (even against patient preferences)	3.49	3.16	4.17	0.047
	**Physician** (while considering patient preferences)	56.33	54.74	60.42	
	**Patient** (while considering physician advice)	34.93	31.58	34.38	
	**Patient**, even when disagreeing with physician recommendation	5.24	10.53	1.04	

**Perceived patient autonomy** was evaluated by two items: taking the initiative to seek information on medical issues (5.6), and general understanding of health issues (5.54), with no significant differences between the subpopulations. Another aspect of autonomy was the respondents’ perception of patients’ freedom to choose whether to follow instructions given by their physician. Two distinct perceptions of freedom of choice emerged. First, over half of the respondents (53.3%) believed that patients would like to be free to decide whether to follow their physician’s instructions, meaning autonomous action. Second, 41.4% believed that patients should fully comply with their physician’s instructions as part of the patient–caregiver dialogue. No significant differences between the respondent subpopulations were found.

The perceived **ability** of patients to act was evaluated by three items: proactive health literacy (mean 5.6), awareness of health topics, and appropriate assessment of the accuracy (mean 5.5), relevancy, and credibility of information given (mean 5.1). No statistically significant differences between the subpopulations were observed.

Most respondents (98%) agreed with the statement that knowledge enables decision-making but that different attention is given to different topics: the respondents believed that patients would focus more on information about treatment opportunities, prognosis, and daily consequences (3.99, 3.98, and 3.72, respectively, on a 1–5 scale) than about innovation (3.57) and the etiology of their disease (3.38) (p = 0.0768, 0.0816 respectively). Once again, no difference between the subpopulations was observed.

The respondents ranked physicians’ accountability very high (8.84) when asked about the responsibility of physicians to ensure that patients understand and comply with caregiving requirements. No significant differences between Hebrew and Arabic speakers were found.

To capture the respondents’ perspectives on the decision-making mechanism, a set of scenarios was introduced (Table 2). Most respondents (91%) preferred a shared decision-making mechanism. A majority (56.33%) stated that physicians should consider the patient’s preferences when making a decision whereas 35% stated that the patient should decide on the basis of the physician’s advice. Only 5.24% of the participants believed that the patient alone should decide, while 3.49% stated that the physician should decide how the patient should be treated even if the patient opposes this decision. A greater proportion of Arabic-speaking respondents than of Hebrew speakers believed that **the physician** should lead the decision while considering the patient’s preferences (60.4% vs. 54.7%, respectively) or that **the patient** should decide while considering physician’s advice (34.4% vs. 31.9%, respectively, p = 0.047). Interestingly, only 1% of Arabic speakers, as against 10.5% of Hebrew speakers, perceived that patients should make the choice even if it clashes with the physician’s choice.

#### Attaining equity among medical teams

To assess standpoints on the importance of possible determinants of equity, seven determinants were identified and the participants were asked to rank their importance from 1 (= less important) to 10 (= highly important): gender, age, place of residence, seniority (i.e., respecting the elderly as leaders of society), having a supportive family (as a network for convalescence), patient’s level of education, and affordability of healthcare services (Table 3).

**Table 3:** Junior Medical Managers’ scoring of the relative importance of determinants of equity gaps in healthcare

	Scoring* by determinants and subpopulation			
Elements	Total	Hebrew- speaking participants	Arabic-speaking participants	P value
Gender	8.59	8.68	8.48	0.583
Seniority/elderly	8.22	8.28	8.24	0.852
Supportive family	7.52	6.89	8.00	0.0009
Age	7.35	7.12	7.43	0.403
Affordability	6.98	6.61	7.68	0.003
Place of residence/ periphery	6.83	6.32	7.48	0.0005
Education	6.03	5.29	6.74	0.0000

*1–10 scale

We found that the most important determinants of equity among JMM were gender (scored 8.59) and being a senior citizen (8.22). Age per se scored lower (7.35), similar to having a supportive family (7.52). Affordability of healthcare services in the absence of public funding scored 6.98, place of residence (center vs. periphery) 6.83, and level of education 6.03. No differences between Hebrew and Arabic speakers were found.

Culture-related differences between the subpopulations were found in four determinants: Arabic-speaking respondents gave *level of education* a higher score in attaining equity (6.74) than did Hebrew speakers (5.29, p = 0.0000), considered *supportive families* important in attaining equity (8.00 and 6.89, respectively, p = 0.0009), and assigned importance to *affordability* (7.68 and 6.61, respectively, p = 0.003), and *place of residence* (7.48 and 6.32, respectively, p = 0.0005).

### The econometric model

The newly developed econometric model shows that the degree of trust in the healthcare system is higher among Jewish JMM than among Arab JMM, with no significant difference between men and women. In addition, age was not found to contribute significantly to the level of trust attributed to the system by Junior Medical Managers (Table 4).

**Table 4:** Econometric model for trust equation. Dependent variable: trust in the healthcare system (OLS regression model)

Variable	Coefficient	Std. error	p. value
Jewish	1.006026	.3706687	0.007
Female	.0748929	.1622813	0.645
Age	− .0506526	.1457415	0.729
Economic	.0951175	.0396357	0.017
Economic*Jewish	− .1023844	.0514704	0.048
Physician	.3820679	.2334182	0.103
Physician*Jewish	− .5792597	.298071	0.053
Constant	5.871049	.2867736	0.000

R-squared = 0.214, N = 216.

However, it turns out that economic considerations (such as physicians’ awareness of patients’ insurance coverage and economic status) bolster people’s trust in the healthcare system, but the contribution of these considerations to the level of trust is significantly higher (and positive) among Arab JMM than among Jewish JMM (which is negative).

Furthermore, it was found that making medical decisions by the attending physician increases trust among JMM in general. Jewish JMM, however, believe that making medical decisions by the attending physician and not by patients themselves decreases the trust that they, the JMM, attribute to the healthcare system.

## Discussion

Although patient-centeredness is an emerging value for meaningful health strategy in many health systems, it is often reported through the viewpoint of policymakers. Our aim was to understand how patient centeredness is perceived by JMM. These junior managers see patients daily and cope with gaps that these meetings incur.

Initial standpoints of the entire sample revealed two complementary observations that many of the participants share: over half encourage the patient to take action while over 40% want the patient to comply with doctor’s instructions, possibly reflecting patient–doctor trust beyond indecisiveness. Our assumption is that caregivers encourage patient autonomy but still consider themselves accountable for patients’ health and wellbeing.

We hypothesized that cultural differences between JMM from Arab and Jewish ethnicities may influence the perception of equity even more than geographic distance.

Our findings suggest that JMM perceive patients’ gender and age as having no influence on physicians’ attitudes. Participants from the minority subgroup, however, believe that healthcare professionals prefer educated patients or those with supportive families. All respondents perceive patients as wishing to be more involved in medical decisions, yet Arabic-speaking respondents believe that patients tend to rely on physicians’ viewpoint while Hebrew speakers perceived that patients wish to lead medical decision by themselves.

The beliefs and standpoints of healthcare professionals, like all individuals, stem primarily from their personal background influenced by cultural perceptions. Here we aimed to observe the balance between individual perception and professional attitude.

No significant differences between Arabic and Hebrew speakers were observed in the perception of core healthcare-system elements (i.e., trust in the healthcare system as a public provider, sense of accessibility of care, physician accountability, and physician awareness of patients’ economic status or insurance coverage).

Using a novel econometric model, we found that our participants believe physician awareness of patients’ economic status and level of insurance coverage amplify patients’ trust. This may be explained by the fact that physician awareness reflects additional personal interest in patients’ status. This perception, stronger and more positively expressed by Arab JMM, may be secondary to the lower economic status of the general Arab population. The role of the physician in leading the decision was reported to increase trust in general yet Jewish JMM believe it may diminish trust, possibly because caregivers tend to overrule patients’ preferences in cases of indecisiveness.

Interestingly, our findings revealed cultural based differences in perceptions between the two ethnic subgroups of JMM, shedding light on perceived manifestations of discrimination in the healthcare system. Similarly, the existing knowledge in the literature regarding health perceptions and behavior of minorities points to a range of racism and discrimination, from refusal to accept treatment from an Arab nurse, through verbal abuse, up to physical violence [36, 37]. Future studies focusing on in-depth interviews with JMM, we believe, will promote a deeper understanding of health equity and related features.

As previously reported [38, 39], trust in the physician is greater than trust in the healthcare system. Our respondents, however, ranked trust and equity unexpectedly low even though they work in a publicly funded healthcare system that is based on the principle of justice and equity [40]. This contradicts the findings of another study that found a positive effect of an increased share of public funding on services utilization [41].

The JMMs perceived patients as not fully able to take care of their own health, with no statistically significant difference between the two subpopulations. This can be explained by a similar national perspective among all citizens toward major issues of equity in health regardless of a wide positive list of publicly funded healthcare services.

Analyzing the contrasting traditional concepts of autonomy and physician paternalism, we obtained two major findings. First, the participants strongly supported the concept of a patient–physician partnership, stating they prefer shared decision-making. More than half, however, stated that the physician should lead the decision-making process while considering the patient’s preferences, whereas only a third stated that the patient should lead this process while consulting with the physician. Arabic-speaking respondents perceived even more that patients prefer to rely on physicians. Similar results were observed among the general public in a survey based on similar principles [42]. These answers may correlate with the respondents’ perception that patients have little **ability** to act. Interestingly, our findings reflect the changing paradigm and evolution in modern healthcare systems toward shared patient–doctor decision-making. Traditionally, medical staff owned the knowledge that dominated caregiving decisions. The current study provides a glance at the future patient–caregiver encounter; in which the patient shares thoughts and dilemmas while the physician reacts in accordance with the patient’s preferences, promoting more personalized patient-centered care. This concept of shared dialogue has not been fully attained both nationally and worldwide and the differences between the subpopulations of caregivers, shown in this study, may assist the healthcare leadership to better adapt to the new reality by educating medical staff properly.

All participants rated gender and social seniority as important for equity, finding no barriers to equality in patient care. Demographic and economic determinants (patient’s education, place of residence, and affordability) scored lower, meaning wider gaps in achieving equity in health. Moreover, Arabic-speaking JMM strongly believe that caregivers may more willingly approach patients who are well educated, have supportive families, live in central regions, and can afford to pay for care, whereas Hebrew-speaking JMM did not report the existence of disparities in these respects.

It has been reported in previous studies that place of residence and affordability of treatment are associated with health inequity and gaps in health outcomes [43–47]. Interestingly, in our research, both subpopulations ranked seniority similarly. The explanation may be that both subpopulations regard the elderly as a vulnerable group or the group most burdened by morbidity, or that seniors should be honored due to their experience and contribution to society.

### Limitations of the study

The study has several limitations that stem from our targeting of a specific group of (JMM) which is an interesting study group: First, this is a relative healthy and educated group with aspirations to advance in workplace and become senior executives, which may possibly lead to a more systematic approach and perspectives and may not necessarily represent perspectives of less educated individuals or those of low SES (Our respondents were knowledgeable about medicine and more familiar with the healthcare system than are patients, who usually face more barriers in obtaining care). Second, this group still holds daily interactions with patients, exposing them to the challenges of the Israeli healthcare, which may affect their responses.

The strengths of our study emerge from our specific sample of JMM, which demonstrates a unique socioeconomic perspective: although the Israeli healthcare system is publicly funded, the respondents’ perceptions on social determinants, especially patients’ SES and ability to pay for healthcare services, contribute importantly to their viewpoint on equity. Accordingly, we realized that interventions meant to close equity gaps require a personalized approach rather than “one size fits all” solutions. An additional strength of having conducted the survey among JMM is the increased awareness of these respondents to inequity among patients with different characteristics. The fact that our respondents represented three different districts is a platform for nationwide diffusion of awareness.

## Conclusions

Healthcare professionals at all levels, as well as healthcare providers worldwide, still consider patients only partly able and willing to lead decisions about their own health. Although patients’ autonomy and shared decision-making are growing, caregivers perceive patients as deeply reliant on physicians, especially among weaker populations.

Patient-centeredness is a fundamental principle in healthcare that should be implemented bottom-up as well as top-down in a transparent nationwide manner. This study revealed the standpoints of JMM—a distinct population of key players in the healthcare arena—from the two major ethnicities in Israel. This population of healthcare professionals can point out gaps in health equity and barriers to the implementation of a patient-centeredness strategy essential for the patient–physician encounter. Moreover, a culture-targeted comparison is a useful tool in identifying central themes for interventions. As expected, gaps in perceptions still exist among healthcare professionals. Analyzing stakeholders’ perceptions at all levels may enhance the understanding of providers, healthcare professionals, and the public of patients’ actual ability and willingness to participate in medical decisions.

The viewpoint of any person—patient, caregiver, or member of the public—is composed of experience, personal beliefs, and cultural values. Here, we wished to understand what influences the standpoint of caregivers more—personal background or professional principles. Witnessing the recent narrowing of social gaps between Arab and Jewish healthcare, we expected to find minimal differences in our participants’ responses in the current survey. Our research, however, shows that many gaps still exist among the young generation of healthcare workers. This is the exact point of influence at which JMM should be educated in bridging the gaps and serving as agents of change not only in the medical environment but in society at large.

We wish to inspire these JMM to use their healthcare assets to increase equity among their patients by enhancing their autonomy and freedom to choose their care instead of employing top-down interventions such as health-promoting activities. We believe that the empowerment of these JMM may create a ripple effect, yielding a bottom- up perception of equity and initiating change.

## Data Availability

The datasets used and analyzed in the study are available from the corresponding author by reasonable request.

## List of abbreviations

JMM: Junior medical managers
SES: Socioeconomic status
HL: Health literacy
